# Regeneration competent satellite cell niches in rat engineered skeletal muscle

**DOI:** 10.1096/fba.2019-00013

**Published:** 2019-11-13

**Authors:** Malte Tiburcy, Alex Markov, Lena K. Kraemer, Peter Christalla, Margret Rave‐Fraenk, Henrike J. Fischer, Holger M. Reichardt, Wolfram‐Hubertus Zimmermann

**Affiliations:** ^1^ Institute of Pharmacology and Toxicology Georg‐August University Göttingen Göttingen Germany; ^2^ DZHK (German Center for Cardiovascular Research), partner site Göttingen Göttingen Germany; ^3^ Department of Radiation Therapy and Radiooncology Georg‐August University Göttingen Göttingen Germany; ^4^ Institute for Cellular and Molecular Immunology Georg‐August University Göttingen Göttingen Germany; ^5^ Present address: Institute for Immunology Medical Faculty RWTH Aachen University Aachen Germany

**Keywords:** Drug screening, Muscle repair, Notch‐signaling, Satellite cell, Skeletal muscle, Tissue engineering, Wnt‐signaling

## Abstract

Satellite cells reside in defined niches and are activated upon skeletal muscle injury to facilitate regeneration. Mechanistic studies of skeletal muscle regeneration are hampered by the inability to faithfully simulate satellite cell biology in vitro. We sought to overcome this limitation by developing tissue engineered skeletal muscle (ESM) with (1) satellite cell niches and (2) the capacity to regenerate after injury. ESMs contained quiescent Pax7‐positive satellite cells in morphologically defined niches. Satellite cells could be activated to repair (i) cardiotoxin and (ii) mechanical crush injuries. Activation of the Wnt‐pathway was essential for muscle regeneration. Finally, muscle progenitors from the engineered niche developed de novo ESM in vitro and regenerated skeletal muscle after cardiotoxin‐induced injury in vivo. We conclude that ESM with functional progenitor niches reminiscent of the in vivo satellite cell niches can be engineered in vitro. ESM may ultimately be exploited in disease modeling, drug screening, or muscle regeneration.

**Abbreviations**
ESM: Engineered Skeletal Muscle
GSI: Gamma‐secretase inhibitor
TA: Tibialis anterior
TT: Twitch tension

## 1 INTRODUCTION

Satellite cells constitute the major stem cell type in skeletal muscle.^1^ Recent studies using transgenic approaches have unequivocally established Pax7‐positive satellite cells as the major source of regeneration in adult skeletal muscle.^2^, ^3^ After skeletal muscle damage satellite cells break quiescence, proliferate and enter the myogenic differentiation program leading to the formation of new muscle fibers, which are characterized by expression of immature myosin isoforms.^4^, ^5^, ^6^ Given these regenerative properties, satellite cells have been suggested as a potential cell therapeutic agent in severe skeletal muscle diseases.^7^ However, implantation of in vitro propagated “satellite cells” has not been overly successful in treating muscular dystrophies.^8^, ^9^ The lack of regeneration described in these studies may be in part attributed to the irreversible activation of satellite cells under standard culture conditions, leading to differentiation into myoblasts and their subsequent fusion into multinucleated myotubes.^10^, ^11^ Only when satellite cells in their muscle fiber niche were transplanted, repopulation of the endogenous niche and widespread muscle regeneration were observed in a mouse model of muscular dystrophy.^1^ In an attempt to better retain the satellite cell phenotype under defined in vitro culture conditions, pliant tissue culture substrates have been introduced to create an artificial niche.^12^, ^13^, ^14^ Data from these studies suggest that biomechanical cues provided by the extracellular environment are important regulators of satellite cell biology.

Tissue engineering adds a third dimension to classical monolayer culture models with the aim to offer more “physiological” conditions and to enhance maturation toward an adult state.^15^, ^16^, ^17^, ^18^ Tissue engineering of skeletal muscle from rodent and human cells has been introduced roughly 20 years ago 19 with implications in drug screening, gene therapy, and tissue replacement therapy.^20^, ^21^, ^22^, ^23^, ^24^ Ideal tissue engineered skeletal muscle should display all characteristic morphological (ie for example fusion of myotubes to form a true syncytium) and functional (ie for example tetanic contractions upon high frequency stimulation) properties of native skeletal muscle.^24^, ^25^, ^26^ It should moreover contain functional satellite cell niches capable of muscle regeneration if activated by injury as this would allow to study muscle regeneration in the dish.^27^

Here, we demonstrate that satellite cells from engineered skeletal muscle (ESM) are capable of regenerating muscle in vitro and in vivo. Satellite cells respond to pharmacological interventions demonstrating the principal utility of ESM in screens for regeneration inducing drugs in vitro.

## 2 MATERIALS AND METHODS

### 2.1 Isolation and culture of rat primary skeletal myocytes

Adult Wistar rats (Charles River) were euthanized in CO_2_ anesthesia by cervical dislocation. Organ harvest was approved by the Niedersächsisches Landesamt für Verbraucherschutz und Lebensmittelsicherheit (LAVES: AZ 10/13). For one series of ESM skeletal muscle from both hind limbs (total 20‐30 g) of one Wistar rat (6‐8 weeks old) was collected in basal medium (DMEM/F12, 5% fetal bovine serum [FBS], 2 mmol/L glutamine, 100 U/ml penicillin, 100 µg/ml streptomycin) on ice. Connective tissue and tendons were removed and the muscle was minced to a fine slurry. This was digested for 90 min at 37°C using 2.5 mg/ml Collagenase II (Biochrom) and 20 µg/ml DNAse I (Calbiochem) in basal medium. The tissue suspension was subsequently passed through filters with gradually decreasing mesh size (250 100 40 µm, BD Biosciences). The cell suspension was then preplated for 1 hour in basal medium on cell culture dishes to reduce the number of fibroblasts. The non‐attached cells were resuspended in proliferation medium (basal medium supplemented with 10 ng/ml bFGF; PeproTech), 10 ng/ml EGF (PeproTech), Insulin‐Transferrin‐Selenite (ITS) + X (100x; Invitrogen) and expanded for 5 days on uncoated flasks without further passaging. For implantation experiments transgenic Lewis rats with ubiquitous expression of eGFP were used for skeletal myocyte preparation.^28^

### 2.2 Generation of engineered skeletal muscle (ESM)

ESMs were generated using a modification of our previously published cardiac muscle engineering protocol.^29^, ^30^, ^31^ Briefly, culture (5 days) expanded primary skeletal muscle cells (1.25x10^6^ total cells/ESM), extracellular matrix (0.4 mg rat tail collagen, 10% Matrigel^®^), and concentrated culture medium (2xDMEM, 20% horse serum, 4% chick embryo extract, 4 mmol/L glutamine, 200 U/ml penicillin, 200 µg/ml streptomycin) were mixed and poured into circular molds (inner/outer diameter: 4/8 mm; height: 5 mm; volume: 450 µl). After incubation for 1 hour at 37°C and 5% CO_2_, ESM culture medium was added (DMEM [Biochrom], 10% horse serum, 2% chick embryo extract, 2 mmol/L glutamine, 100 U/ml penicillin, 100 µg/ml streptomycin and 1x ITS + X). Medium was changed after 24 hours and then every other day. ESMs condensed within 5 days to form a mechanically robust ring‐shaped tissue construct and were then transferred onto custom made holders for continuous culture at 110% of slack length for additional 7 days.

### 2.3 Myoblast culture

For 2D culture primary myogenic cells were plated on the ESM matrix mixture (Collagen type I plus Matrigel^®^; diluted 1:50 in PBS) and cultured in parallel for 12 days in ESM culture medium (see above).

### 2.4 Contraction measurements

Twitch tension was measured under isometric conditions in continuously gassed (95% O_2_/5% CO_2_) modified Tyrode's solution at 37°C as described before.^30^, ^32^ L_max_ (ie length of maximal twitch tension [TT] development) was determined at 2.5 Hz stimulation frequency (field stimulation, pulse width 4 ms, voltage 10% above threshold) by increasing resting length (L_0_) until TT reached maximal values. Maximal force response was assessed at 2.5, 5, 20, 40, 60, 80, 100 Hz. Maximal TT was determined under 80 Hz tetanic stimulation.

### 2.5 Measurement of elastic modulus

The Young's modulus (E) of ESM was measured at 37°C in Tyrode's solution in thermostatted organ baths. Stress‐strain curves were derived by repeatedly increasing the length of the ESM starting from slack length. Passive force was recorded after it had reached a steady‐state. The stress (kPa) was calculated from the passive force (mN) and the ESM cross‐sectional area (mm^2^). Strain was calculated by dividing ESM length change (delta) by the ESM slack length. The Young's modulus was derived from the slope of the stress‐strain curve at the range of physiological strain (0%‐10% length change).

### 2.6 Immunocytochemistry

ESMs were fixed in 4% formaldehyde or 70% ice cold ethanol overnight at 4°C. Primary skeletal muscle was fixed overnight in 4% formaldehyde. For cryosectioning ESM/skeletal muscle were immersed in 30% sucrose in PBS and embedded in OCT compound. Sections were cut at 10 µm thickness using a Leica Cryotome (CM3050 S). For whole mount staining of ESM, permeabilization and blocking was achieved by overnight incubation in phosphate buffered saline (PBS), 5% goat serum, 1% bovine serum albumin (BSA), and 0.1% Triton‐X. The same buffer was used for the primary and secondary antibody incubation. Labelling of Pax7 (Developmental Studies Hybridoma Bank, DSHB), Ki67 (Pierce), embryonic myosin heavy chain (Vector Laboratories), tropomyosin and laminin (both Sigma), m‐cadherin and caveolin‐1 (both Santa Cruz), caveolin‐3 (BD Biosciences), ED1/ ED2 (AbD Serotec), and prolyl‐4‐hydroxylase (Millipore), and GFP (Invitrogen) was performed overnight at 4°C followed by several washes with PBS. Incubation with appropriate Alexa fluorochrome labelled secondary antibodies (Invitrogen, 1:1,000) was performed overnight at 4°C. Where indicated labelling of f‐actin and nuclei was performed using Alexa 488‐conjugated phalloidin 1:60 (Invitrogen) and 4',6‐diamidino‐2‐phenylindole (DAPI; 1 µg/ml) in parallel to secondary antibodies. Plated cells were fixed in 4% formaldehyde for 10 minutes at room temperature. After blocking for 30 minutes, primary antibodies were added overnight at 4°C. After three washes with PBS, appropriate secondary antibodies were applied for 1 hour at room temperature. Cells were washed and mounted in Fluoromount‐G (Southern Biotech). All stainings were imaged on a Zeiss LSM 710/NLO confocal microscope. Quantification of labelled cells was performed by imaging at least three randomly chosen focal planes per ESM of at least three different series. Cell counting was done using ImageJ cell counter tool.

### 2.7 Flow cytometry

Cells were fixed in 4% formaldehyde for 10 minutes. Staining for Pax7 (DSHB), desmin (Sigma), and ED1/ ED2 (AbD Serotec) was performed at 4°C for 45 minutes. Appropriate secondary antibodies were applied for 30 minutes at room temperature. Isotype controls were prepared in parallel. Samples were run on a LSRII cytometer and analysed with Facs Diva software (BD Biosciences).

### 2.8 Quantitative real time PCR

Snap‐frozen ESMs were lysed in RNA extraction buffer (Trizol, Invitrogen) using the Tissue lyser device (Quiagen, 25 strokes/second). RNA was extracted according to manufacturer's recommendations followed by reverse transcription of 1 µg RNA with Superscript II kit (Invitrogen). Quantitative PCR was performed using 50 ng of cDNA as template, TaqPolymerase Master Mix (Applied Biosystems), TaqMan^®^ primer/probe sets, and a 7900 HT Applied Biosystems Cycler. The following primer/probe sets were used:

**Table:** 

	
GAPDH forward GAPDH reverse	AACTCCCTCAAGATGTCAGCAA CAGTCTTCTGAGTGGCAGTGATG
GAPDH probe (5’‐Fam/3’‐Tamra‐labeled; 5’3’)	ATGGACTGTGGTCATGAGCCCTTCCA
Pax7 forward	TATCCCATGGCTGTGTCTCC
Pax7 reverse	ATTTCCCAGCTGAACATTCC
Pax7 probe (5’‐Fam/3’‐Tamra‐labeled; 5’3’)	CAAGCCCAGACAGGTGGCGACT
MyoD forward	CTACGACGCCGCCTACTAC
MyoD reverse	GGAGATGCGCTCCACTATG
MyoD probe (5’‐Fam/3’‐Tamra‐labeled; 5’3’)	AGTGAGGCGTCCAGCGAGCC
Myh3 forward	TTCTTGTGGATGGTCACTCG
Myh3 reverse	ACGAACATGTGGTGGTTGAA
Myh3 probe (5’‐Fam/3’‐Tamra‐labeled; 5’3’)	TGAGTATAACAGCCTGGAGCAGCTGTGC
Myh1,2,4 forward	GCAGGCTGAGATCGAGGA
Myh1,2,4 reverse	TGGATCTGGGAAATGTCTGTC
Myh1,2,4 probe (5’‐Fam/3’‐Tamra‐labeled; 5’3’)	CACACCCAGAACACCAGCCTCATCA
Myh7 forward	GCCTACAAGCGCCAGGCT
Myh7 reverse	CATCCTTAGGGTTGGGTAGCA
Myh7 probe (5’‐Fam/3’‐Tamra‐labeled; 5’3’)	TTCATTCAGGCCCTTGGCGCCAAT
Myh8 forward	AGGGAGGGAAGCATATCCAC
Myh8 reverse	AGGATCTTTCCCTCCTCGTG
Myh8 probe (5’‐Fam/3’‐Tamra‐labeled; 5’3’)	CTGCTTTAGAGGAAGCAGAGGCATCTCT

### 2.9 ESM injury models

To induce skeletal muscle injury, cardiotoxin (CTX: 25 µg/ml, Latoxan) was applied for 24 hours. Mechanical (“crush”) injury was induced with fine anatomical forceps (tip width: 1 mm). 3 short, full squeezes (one second each) were applied to one arm of the ESM yielding a reproducible local injury. To irreversibly inactivate satellite‐like cells, ESMs were taken off the holders and subjected to a single dose of 30 Gy (applied in ~ 13 minutes) irradiation using an STS Biobeam 8000 (Germany) gamma irradiator.

### 2.10 Measurement of adenylate kinase (AK) activity

Cell lysis was assessed by measuring the release of adenylate kinase into the culture medium. Activity of AK was determined in culture medium supernatant after 24 hours of cardiotoxin treatment utilizing the Toxilight assay (Lonza) and a multiwell plate reader (FlexStation 3, Molecular devices). Values were calculated relative to full lysis induced by ToxiLight 100% lysis reagent.

### 2.11 Drug testing

ESM were injured with CTX on culture day 12 and treated with the following substances or vehicle (DMSO 0.1% or water) for 7 days from culture day 14‐21:0.5 µmol/L γ‐secretase inhibitor (GSI, Calbiochem), 5 µmol/L IWR‐1 (Calbiochem), 1 µmol/L CHIR99021 (Stemgent), 100 nmol/L Oxytocin (Sigma‐Aldrich), and 100 nmol/L Angiotensin‐II (Sigma‐Aldrich).

### 2.12 Satellite cell isolation from ESM

To isolate single mononuclear cells, ESMs were incubated in collagenase 1 solution (Sigma‐Aldrich, 1 mg/ml) with 1:10 (v:v) dispase (BD Biosciences) and 20 µg/ml DNAse (Calbiochem) in calcium‐containing PBS at 37°C for 60 minutes. Cells were then resuspended in DMEM/F12, 15% fetal bovine serum [FBS], 2 mmol/L glutamine, 100 U/ml penicillin, and 100 µg/ml streptomycin, mechanically separated, strained (35 µm cut‐off; Miltenyi Biotec) and used without further in vitro expansion for either secondary ESM generation in vitro or implantation in vivo.

### 2.13 In vivo regeneration model

Animal experiments were approved by the Niedersächsisches Landesamt für Verbraucherschutz und Lebensmittelsicherheit (LAVES: AZ 11/0581). The hind legs of RNU nude rats (Charles River) were irradiated with 40 Gy using a RS 225 X‐Ray Research System (Gulmay Medical Systems, UK). 24‐72 hours after irradiation, the right TA muscle was injected with 100 µl of 20 µmol/L CTX to induce muscle damage. 72 hours after CTX‐induced damage, the myogenic cell suspension from 2D or ESM culture was injected into the injured muscle (5x10^5^ cells in 100 µl DMEM/F12 medium) using a 27 G needle.

### 2.14 Statistical analysis

Data are presented as mean ± standard error of the mean (SEM). Statistical differences were determined using unpaired Student's t test or non‐parametric Mann‐Whitney test (two‐tailed) or ANOVA followed by appropriate *post hoc* test as indicated. A p value < 0.05 was considered statistically significant.

## 3 RESULTS

### 3.1 Engineered skeletal muscle with differentiated morphology and functionality

**Figure 1:**
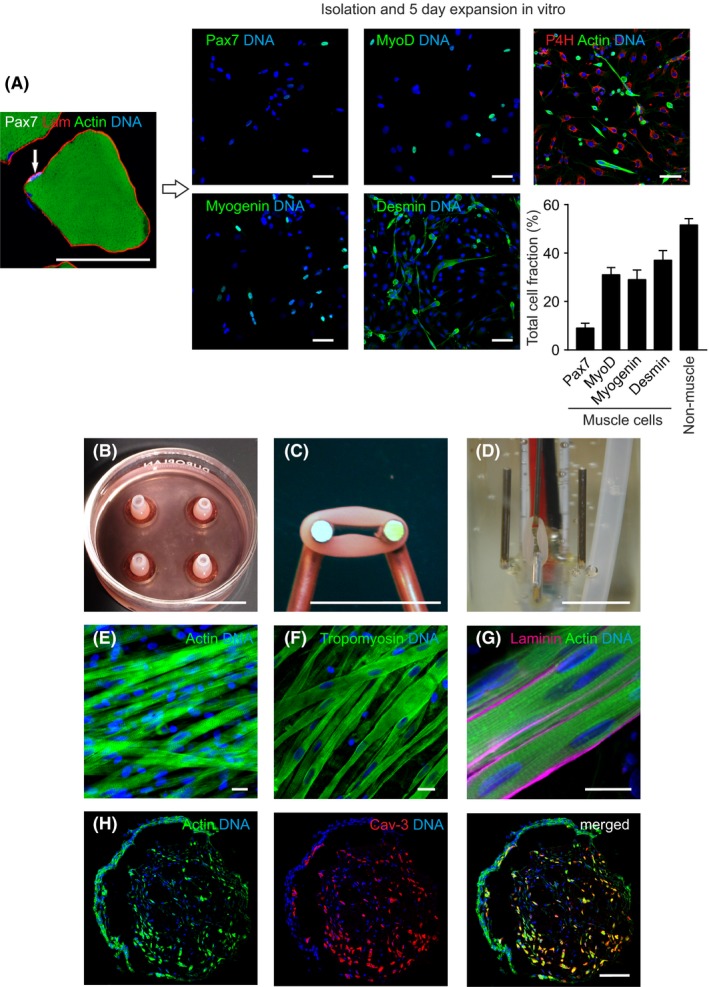
Generation of engineered skeletal muscle from primary skeletal muscle cell isolates. **A**, Satellite cell niche in vivo. Cross section of adult rat vastus lateralis muscle. Arrow: Pax7‐positive satellite cell. Pax7: white, laminin: red, actin: green, nuclei: blue. Isolated cells were expanded for 5 days, characterized and quantified by immunostaining for Pax7, MyoD, Myogenin and Desmin (marker in green, Nuclei: blue). Non‐muscle cells were stained for prolyl‐4‐hydroxylase (P4H), a rat fibroblast specific marker 29, 30 in primary skeletal muscle cell isolates. P4H: red, Actin: green, Nuclei: blue. Quantification of respective marker‐positive cells in percent of total cell fraction. **B**, Primary skeletal muscle cell isolates were submerged in collagen/Matrigel hydrogels, the mixture was cast in circular molds, and cultured for 5 days to form ESM (a casting mold with 4 ESM in culture is displayed). **C**, Culture on metal holder (uniaxial suspension/loading) for additional 7 days. **D**, ESM in organ bath for functional analyses on culture day 12. **E**, Immunostaining for actin (green), and nuclei (blue) in 12 days old ESM. **F**, Immunostaining for tropomyosin (green), and nuclei (blue) in 12 days old ESM. **G**, Immunostaining for laminin (magenta), actin (green), and nuclei (blue) in 12 days old ESM. **H**, Cross‐section of 12 days old ESM. Immunostaining for actin (green), caveolin‐3 (red), and nuclei (blue).Scale bars: 50 µm (A), 1 cm (B, C, D), 20 µm (E‐G), 100 µm (H)

We isolated skeletal muscle cells from adult Wistar rat hind limb muscle and cultured them in vitro for maximally 5 days to expand cell numbers, but minimize the impact of in vitro culture on cellular properties. The primary muscle cell isolates contained 9 ± 2% Pax7‐positive, 31 ± 3% MyoD‐positive, 29 ± 4% Myogenin‐positive, and 37 ± 4% desmin‐positive myogenic cells (n = 3‐4 preparations), with the remaining non‐muscle cells being predominately prolyl‐4‐hydroxylase‐positive fibroblasts (Figure 1A**, **Figure S1). Engineered skeletal muscle (ESM) was generated from these primary muscle cell isolates, by mixing with solubilized collagen type 1 and Matrigel^™^. This reconstitution mixture was cast into circular molds, which facilitated condensation into mechanically stable circular tissue constructs within 5 days (Figure 1B). We subsequently transferred ESMs onto custom made holders for additional 7 days to maintain them under a defined load (Figure 1C). This procedure yielded contractile (Figure 1D, Movie S1) ESM with morphologically well‐differentiated actin and tropomyosin‐positive muscle fibers lined by a Laminin‐positive basal lamina (Figure 1E‐G). Three‐dimensional reconstitution of optical tissue sections of the whole ESM identified well‐organized and aligned muscle syncytia (Movie S2). Cross sections of ESM demonstrated a homogeneous distribution of muscle cells (identified by muscle‐specific caveolin‐3) throughout the tissue with a denser network of non‐muscle cells lining the outer edge (Figure 1H).

**Figure 2:**
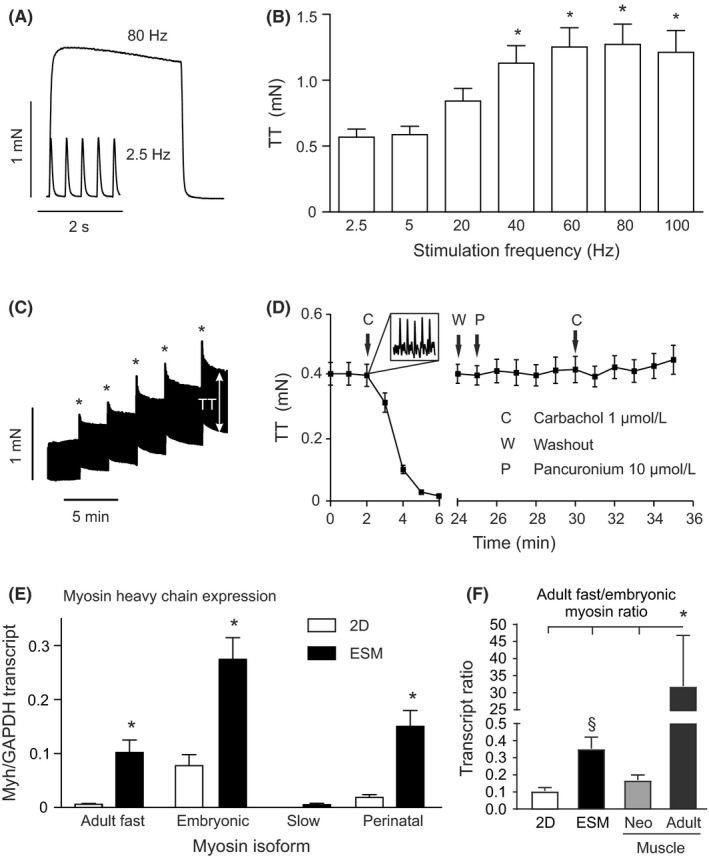
ESM exhibit organotypic functional and molecular properties. **A**, Representative single twitches at 2.5 Hz and tetanus at 80 Hz electrical field stimulation. **B**, Higher stimulation frequencies increase twitch tension (TT) development (max. at 80 Hz). n = 14/group, *P < 0.05 vs. 2.5 Hz by ANOVA and Dunnet *post‐hoc* test. **C**, Positive length‐force‐relationship: increases in muscle length by stretch (asterisks) resulted in higher ESM twitch tension (recorded at 2.5 Hz field‐stimulation; arrow indicates TT). **D**, Depolarizing muscle block by carbachol (left part; inset: transient fasciculations upon addition of carbachol) could be prevented by pre‐incubation with the nicotinergic acetylcholine receptor antagonist pancuronium. **E**, Myosin heavy chain expression in ESM versus 2D culture: fast adult myosin (primers recognize all fast adult myosin isoforms: Myh1, 2, and 4), embryonic myosin (Myh3), slow myosin (Myh7), and perinatal myosin (Myh8) transcript abundance per GAPDH transcript in 2D differentiated myotubes (white bars) and ESM (black bars); n = 8/group, *P < 0.05 2D vs. ESM by unpaired Student's t‐test (two‐tailed). **F**, Ratio of embryonic to adult fast myosin isoform expression in 2D and ESM (n = 8/group) culture compared to neonatal and adult vastus lateralis muscle (n = 3/group). *P < 0.05 by 1‐way ANOVA and Tukey's post‐hoc test; §*P* < 0.05 vs. 2D and neonatal muscle

**Figure 3:**
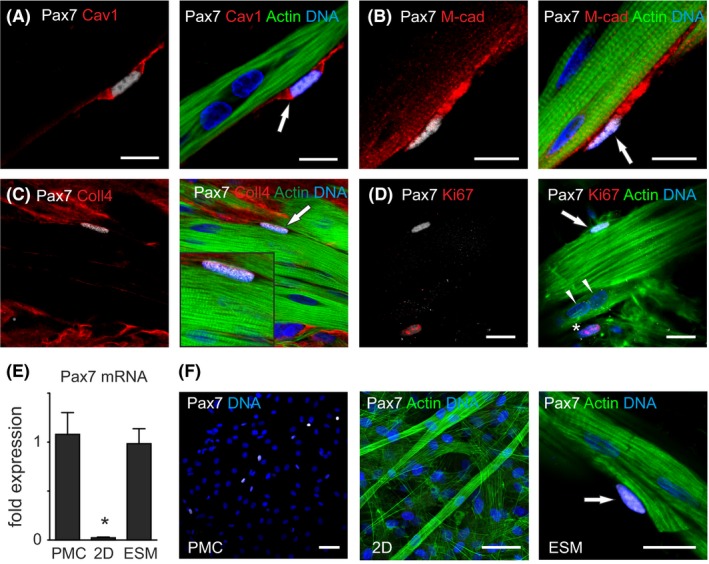
Reconstruction of the satellite cell niche in ESM. **A‐D**, Immunostaining in ESM for common morphological markers that define the *bona fide* satellite cell niche: **A**, caveolin‐1 (cav1, red), **B**, m‐cadherin (m‐cad: red), **C**, Collagen IV (Coll4: red), and **D**, Ki67 (red), Pax7 (white), f‐actin (green), and nuclei (blue). Arrows: Pax7 positive satellite cell niche; arrowheads in (D): Ki67‐negative nuclei in myotube nuclei; asterisk in (D): Ki67 positive nucleus not associated with the satellite cell niche. Scale bars: 10 µm **E**, Pax7 mRNA in primary skeletal muscle cell isolate (PMC) and after 12 days of 2D‐ and 3D (ESM)‐culture. n = 5‐8/group, *P < 0.05 by 1‐way ANOVA and Bonferroni *post‐hoc* test. **F**, Pax7 immunostaining of PMC (left panel) and after 12 days in 2D (middle panel) and ESM (right panel) culture. 2D culture was done on ESM matrix mixture‐coated plates (Collagen‐1, Matrigel^®^, diluted 1:50 in PBS). Arrow depicting Pax7^+^ cell in a typical satellite cell position; ie adjacent to myotube. Pax7: white, f‐actin: green, nuclei: blue. Scale bars: 20 µm

**Figure 4:**
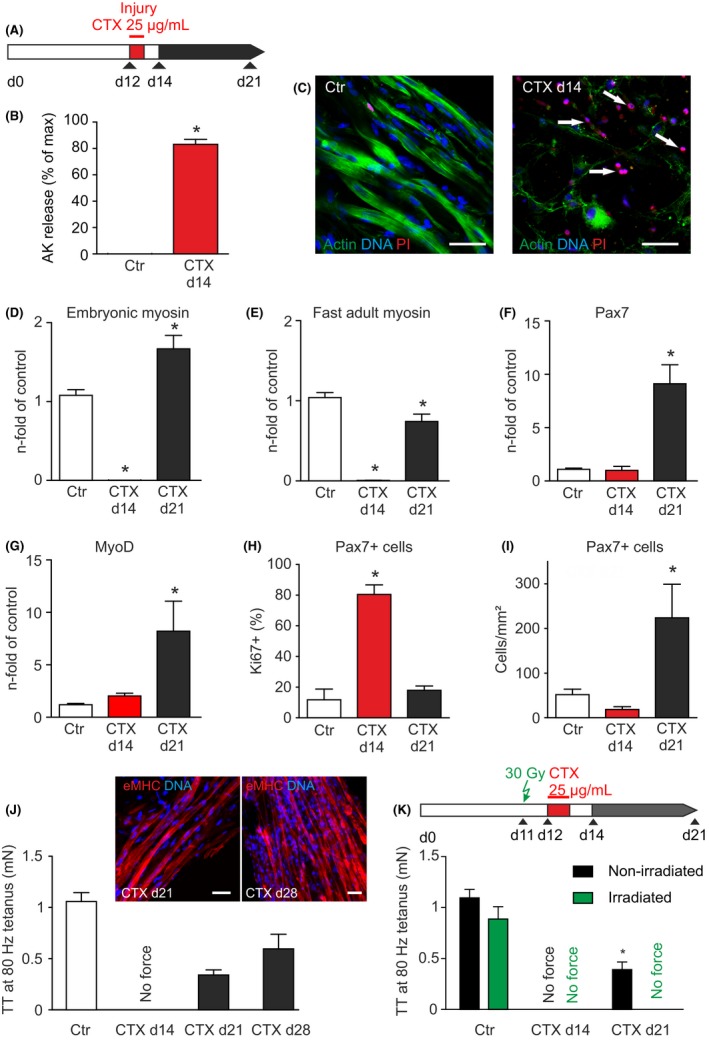
Cardiotoxin‐induced muscle injury and regeneration in ESM. **A**, Schematic of experimental protocol: cardiotoxin (CTX; 25 µg/ml) was applied on ESM culture day 12 for 24 hours. Analyses were performed on culture days 14 (ie 24 hours after CTX‐wash out) and 21. **B**, Adenylate kinase (AK) release into culture medium at indicated time points; n = 8, *P < 0.05 vs. Ctr by unpaired Student's t‐test (two‐tailed). **C**, Propidium iodide (PI) staining of control (left panel) and CTX‐treated (right panel) ESMs. Arrows: PI‐positive nuclei. Scale bars: 50 µm. **D**, Embryonic myosin (Myh3), **E**, fast adult myosin (primers for Myh1, 2, 4), **F**, Pax7, and **G**, MyoD transcripts in CTX‐treated ESMs at culture days 14 (red bars) and 21 (black bars) compared to controls (white bars). Controls in (D), (E), (F), and (G) include corresponding reference values of day 14 and day 21‐ n = 15‐18 (controls); n = 5‐8 (CTX d14, d21), *P < 0.05 vs. Ctr. by ANOVA and Bonferroni *post‐hoc* test. **H**, Percentage of Pax7^+^ cells staining positive for the cell cycle marker Ki67 in control ESMs (white bars) and CTX‐treated ESMs at culture days 14 (red bars) and 21 (black bars), n = 4‐5/group. *P < 0.05 vs. Ctr. by ANOVA and Bonferroni *post‐hoc* test. **I**, Total number of Pax7^+^ cells in control ESMs (white bar) and CTX‐treated ESMs at culture days 14 (red bar) and 21 (black bar), n = 4‐5/group. *P < 0.05 vs. Ctr. by ANOVA and Bonferroni post‐hoc test. **J**, Tetanic force (80 Hz) of control (white bar) and CTX‐treated (for 24 hours on culture day 12, black bars) ESMs on the indicated culture days; n = 14‐16/group (d14, d21), n = 4 (d28); (**inset**) eMHC‐positive muscle cells after CTX treatment at culture day 21 and day 28 (eMHC: red, nuclei: blue, scale bar 20 µm). **K**, Schematic of alternative experimental protocol: one group of ESMs was irradiated with 30 Gy on culture day 11 (ie 1 days before CTX application: 25 µg/ml for 24 h). Measurements of tetanic force at 80 Hz field stimulation were performed on indicated culture days. Tetanic force of non‐irradiated ESM (black bars) and irradiated ESMs (green bars); n = 4/group, *P < 0.05 non‐irradiated versus irradiated by unpaired Student's t‐test (two‐tailed)

Analysis of contractile function in rat ESM under isometric conditions in organ baths (Figure 1F) revealed typical skeletal muscle properties, including (1) tetanic contractions at high stimulation frequency (maximal tetanic force 1.3 ± 0.2 mN at 80 Hz, n = 14; Figure 2A), (2) a positive force‐frequency response (Figure 2B), (3) a positive force‐length relationship (Figure 2C), and (4) depolarizing muscle block induced by the cholinergic receptor agonist carbachol which could be antagonized by the non‐depolarizing, cholinergic receptor antagonist pancuronium (Figure 2D). When normalized to mean muscle cross‐sectional area (CSA) the tetanic force corresponded to a specific force of 21 ± 1 kN/m2 (n = 13). This is about 10% of the specific force of native fast skeletal muscle,^33^ indicating high but not fully functional maturation of muscle fibers within ESM. Enhanced expression of mature myosin heavy chain transcripts (Figure 2E and 2F) in ESM suggested advanced maturation compared to conventional 2D skeletal muscle cell culture. The fast twitch characteristics (time to peak: 43 ± 1 ms, time to 50% relaxation: 47 ± 2 ms, n = 20 ESM) and the absence of slow myosin transcripts provided functional and molecular evidence for the development of fast‐type muscle rather than slow‐type muscle phenotype in ESM and are comparable to day 4 neonatal rat fast muscle.^34^

### 3.2 Engineered skeletal muscle contains muscle progenitor niches

ESMs are composed of differentiated and functionally mature myofibers arranged as true multinucleated muscle syncytia. We next investigated if ESM contain a stem cell pool similar to native muscle. Interestingly, we identified mononucleated cells expressing the satellite cell transcription factor Pax7 in ESM, 77 ± 5% (n = 3 ESM) of which were positioned adjacent to differentiated skeletal myofibers, reminiscent of their typical anatomical location in vivo (Figure 1A; arrow). In addition to Pax7, these cells also expressed other characteristic satellite cell markers, including caveolin‐1 and m‐cadherin (Figure 3A, 3) and were situated beneath a collagen IV (Figure 3C) positive basal lamina, a characteristic of the in vivo satellite cell niche. As further evidence for an in vivo satellite cell phenotype, 88 ± 7% (n = 3 ESM) of these Pax7 cells were negative for the cell cycle marker Ki67, indicative of cell cycle quiescence (Figure 3D). Ki67 staining also indicated that fused myotubes were post‐mitotic (Figure 3D; arrows). Our model therefore seems to provide the appropriate cues to facilitate both, (1) fusion and differentiation of muscle cells into post‐mitotic myotubes and (2) development of a muscle progenitor cell niches reminiscent of the physiological satellite cell niches.

To scrutinize which conditions favor the formation of the niche in ESM we compared Pax7 expression in 3D (ESM) and parallel 2D cultures. Pax7 transcript and protein was clearly detectable in ESM, but not in 2D indicating the maintenance of *bona fide* skeletal muscle progenitor cells in 3D (Figure 3E and 3F). We hypothesized that the main difference between 3D and 2D was the substrate stiffness. Interestingly, muscle syncytia in 12 day old ESM exhibited passive forces of 9.5 ± 1.4 kPa (elastic modulus [E], n = 10), which is similar to the elastic modulus reported for tibialis anterior muscle and which was reported to favor satellite cell “stemness”.^12^

### 3.3 Cardiotoxin‐induced muscle injury and regeneration

We next investigated the response of ESM to muscle injury to scrutinize the functionality of the engineered satellite cell niches. This was firstly tested by inducing muscle damage with cardiotoxin (CTX 25 µg/ml; for 24 hours) on ESM culture day 12 (Figure 4A). On ESM culture day 14 (ie after an additional 24 hours of CTX washout) we noted severe muscle damage with almost complete destruction of skeletal muscle cells in ESM as determined by adenylate kinase release (Figure 4B) as well as f‐actin (to identify myotubes) and propidium iodide (PI; to identify nuclei of dead or dying cells) labeling (Figure 4C; arrows). This massive muscle cell lysis was paralleled by a complete loss of characteristic skeletal muscle transcripts, including embryonic myosin and fast adult myosin (Figure 4D and 4E). Interestingly, Pax7 transcripts remained largely unaffected, suggesting survival of satellite cells under these conditions (Figure 4F).

Enhanced MyoD transcript expression (culture day 14; Figure 4G) provided first molecular evidence for the activation of the satellite cell niche in damaged ESM. This notion was further substantiated by histological analyses demonstrating marked cell cycle activation (Ki67‐positivity) in Pax7 positive cells two days after injury (culture day 14, Figure 4H) and a subsequent increase in Pax7 positive cells at culture day 21 (ie 9 days after injury; Figure 4I). This finding is also in agreement with the concurrent up‐regulation of Pax7 transcripts, observed in ESM on culture day 21 (Figure 4F). As an apparent consequence of satellite cell activation, we observed re‐expression of embryonic and adult myosin genes (Figure 4D and 4E).

Importantly, the net effect of this regenerative response could be robustly assayed by tetanic force measurements. Here we found, consistent with the transcript and protein data, a partial recovery of tetanic force (to 32 ± 5% of initial force, n = 16) 7 days following CTX‐induced complete contractile failure (Figure 4J). The functional regeneration correlated with the morphological evidence of newly formed eMHC‐positive muscle fibers (Figure 4J, inset). Persistent activation of the regenerative potential in ESM resulted in a further normalization of contractile performance after additional 7 days of regeneration (to 56 ± 13% of initial force, n = 4, Figure 4J).

To test whether regeneration of contractile force was indeed related to the activation of satellite cells, we irradiated ESMs with 30 Gy before CTX injury (Figure 4K). This experimental model is widely used to inhibit cell cycle activity and thus endogenous repair capacity in skeletal muscle in situ.^35^, ^36^ In line with the findings from respective experimental animal models, ESMs were not significantly affected by irradiation alone (Figure 4K). However, functional and morphological regeneration 9 days after CTX injury (culture day 21) was completely abolished in irradiated ESMs (Figure 4K**, **Figure S2A). This data suggests that cell division of satellite cells was essential for the muscle regeneration in non‐irradiated ESM.

Considering the important role of macrophages in skeletal muscle regeneration in vivo and in vitro,^37^, ^38^ we asked whether ESM contain a macrophage population. Indeed, we identified a population of ED1 (CD68) and ED2 (CD163)‐positive macrophages that were present in the ESM input cells (Figure S1) and that are likely instrumental in the robust regenerative response seen in ESM (Figure S2B).

### 3.4 Regeneration after mechanical injury

**Figure 5:**
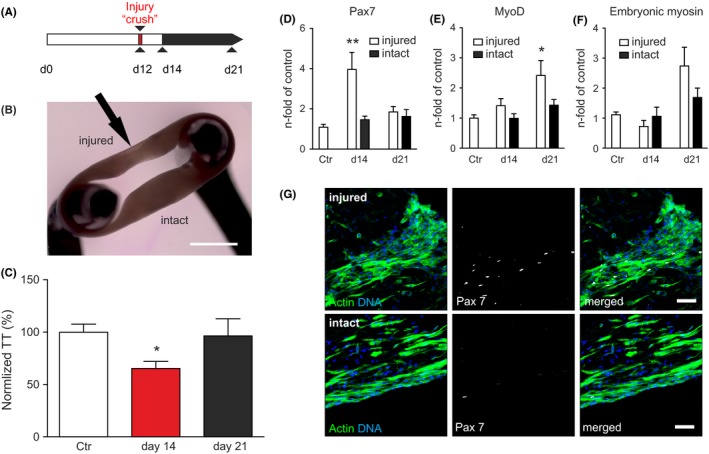
Satellite cell activation and ESM regeneration in response to crush injury. **A**, Schematic of experimental protocol: on culture day 12 one arm of the ESM was squeezed with forceps to induce a defined “crush injury”. **B**, Picture of ESM on stretcher with one arm injured (arrow) and the other arm intact. **C**, Tetanic twitch force of control (culture day 14) and crush injured ESMs on culture days 14 (red bars) and 21 (black bars), *P < 0.05 vs. Ctr. by 1‐way ANOVA with Dunnet's *post‐hoc* test; controls include corresponding reference values from day 14 and day 21 ‐ n = 16 (controls); n = 8 (d14), n = 8 (d21). **D**, Pax7, **E**, MyoD, and **F**, embryonic myosin (Myh3) transcript abundance in the injured arm (white bars) and the intact arm (black bars) at culture days 14 and 21 compared to controls; controls include corresponding reference values from day 14 and day 21 ‐ n = 11‐12 (controls); n = 8 (d14), n = 4 (d21); *P < 0.05 vs. Ctr. by 2‐way ANOVA with Bonferroni's *post‐hoc* test;. **G**, Immune staining for Pax7^+^ cells in injured and intact arms of ESM at culture day 14. Pax7: white; f‐actin: green; nuclei: blue. Scale bars: 1 mm (B) and 20 µm (G)

As CTX induced severe muscle injury, we asked whether a less dramatic and likely physiologically more realistic local mechanical injury would also activate satellite cells in ESM to facilitate skeletal muscle regeneration. By applying repeated mechanical compressions (3 times, one second long) with a pair of fine forceps restricted to one arm of the ESM at culture day 12 we induced a reproducible “crush injury” (Figure 5A and 5B). As opposed to the full contractile failure in response to the CTX injury protocol, we observed only a mild but reproducible deterioration of contractile force 2 days after injury (ie ESM culture day 14) and full recovery 7 days following injury **(**Figure 5C). Notably, the molecular consequences of the regenerative response to the crush injury model were similar as in the cardiotoxin injury model with up‐regulation of Pax7 (4 ± 0.8 fold; Figure 5D) 2 days after injury, followed by MyoD (2.4 ± 0.5 fold Figure 5E) and embryonic myosin (2.7 ± 0.6 fold; Figure 5F) up‐regulation 7 days after injury (n = 4‐7/group). These observations were restricted to the injured “arm” of the ESMs, demonstrating the specificity of the injury response by the engineered satellite cell niche. In line with these findings, we identified an enhanced quantity of Pax7‐positive cells at the site of the simulated crush injury (Figure 5G).

### 3.5 Pharmacological modulation of muscle regeneration in vitro

**Figure 6:**
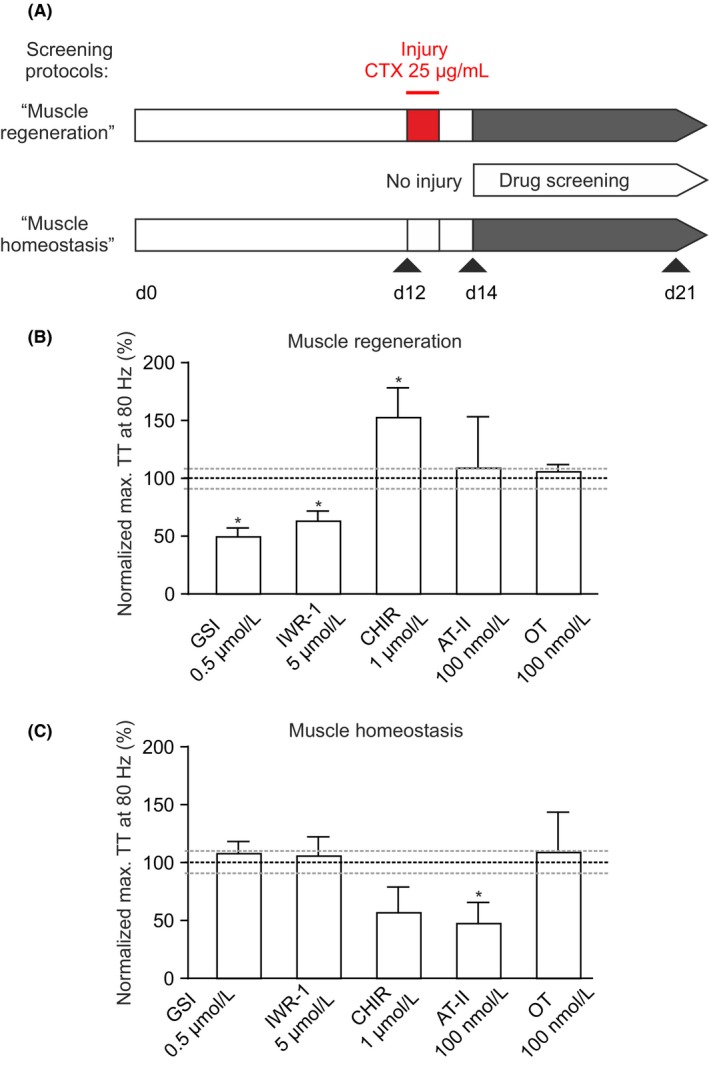
Muscle regeneration enhancing drugs and underlying mode of action identified in ESM. **A**, Schematic of experimental protocol: cardiotoxin (CTX; 25 µg/ml) was applied on ESM culture day 12 for 24 hours followed by drug or vehicle treatment from culture day 14‐21 (“Muscle regeneration”). In parallel the drug effects were tested on uninjured ESM (“Muscle homeostasis”). **B‐C**, 80 Hz tetanic stimulation induced twitch tension (TT) at day 21 of injured **B**, and uninjured **C**, ESM treated with indicated compounds and concentrations (GSI: γ‐secretase inhibitor, n = 5/6; IWR‐1: canonical Wnt inhibitor, n = 12/6; CHIR: Wnt activator, n = 5/3; AT‐II: Angiotensin‐II, n = 3/3; OT: Oxytocin, n = 3/3). TT was normalized to vehicle treated ESM (n = 16/12; mean ± SEM marked by dashed lines). *P < 0.05 vs. vehicle treated control ESM by unpaired Student's t‐test (two‐tailed)

We next tested if muscle regeneration can be modulated in vitro by pharmacological treatment. ESM injured by CTX were treated with substances targeting signaling pathways implicated in skeletal muscle regeneration. Also the effect of the drug was tested in uninjured ESM thereby measuring the effect on muscle homeostasis (Figure 6A). Inhibition of Notch signaling by a γ‐secretase inhibitor (GSI) reduced satellite cell expansion and muscle regeneration (Figure 6B**, **Figure S3) without negative effect on muscle baseline function (muscle homeostasis, Figure 6C). Similarly, inhibition of canonical Wnt signaling (by IWR‐1) had a negative impact on regeneration without effects on muscle homeostasis (Figure 6B,C). These results are consistent with the role of Notch and Wnt signaling described in myogenic regeneration in vivo.^10^, ^39^, ^40^, ^41^ Confirming the essential role of Wnt signaling in muscle regeneration an activator of canonical Wnt signaling (CHIR‐99021) enhanced muscle regeneration (Figure 6B), but muscle function was depressed under basal conditions (Figure 6C). Both Angiotensin‐II and Oxytocin did not have a significant effect on muscle regeneration in ESM (Figure 6B), Angiotensin‐II negatively affected basal muscle homeostasis (Figure 6C). Collectively, these data suggest that ESM can be used to screen for compounds with skeletal muscle regeneration enhancing properties.

### 3.6 ESM‐derived Pax7^+^ satellite cells repopulate the skeletal muscle stem cell niche in vitro and in vivo

**Figure 7:**
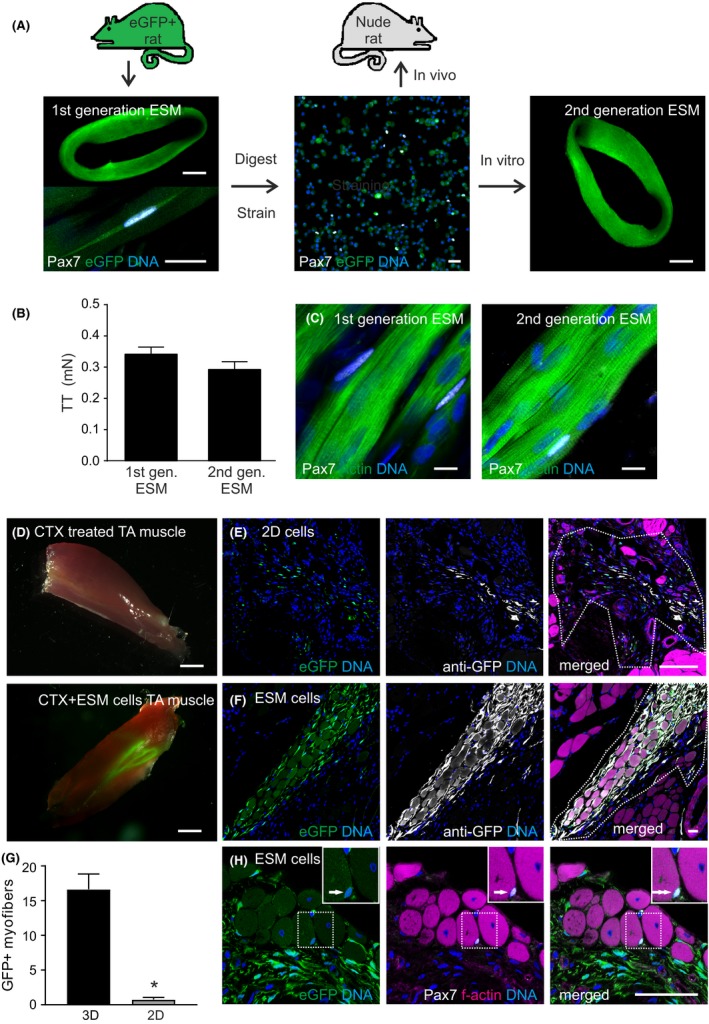
Repopulation of the satellite cell niche in vitro and in vivo*.* **A**, Myofibers with attached Pax7‐positive satellite cells (lower left panel) were isolated from eGFP^+^ ESM (“1st generation ESM”); after enzymatic dispersion and differential straining only mononucleated cells were retrieved (middle panel) and utilized to either generate “2nd generation ESM” or as implant into CTX‐damaged skeletal muscle of regeneration incompetent (by γ‐irradiation) nude rats. Scale bars: 1 mm in full ESM images, 20 µm in lower left, and 100 µm in middle panel. **B**, 80 Hz tetanic stimulus induced witch tension (TT) of parental 1st and 2nd generation ESM (12 days old, n = 3/group). **C**, Immunostaining for Pax7‐positive satellite cells in 1st and 2nd generation ESM (Pax7: white, f‐actin: green, nuclei: blue). Scale bars: 10 µm. **D**, Macroscopic view of explanted nude rat TA muscle 33 days after CTX‐injury (upper panel) and 30 days after implantation of myogenic cells from eGFP^+^‐ESM (lower panel). Note that both images were taken under similar epifluoresence excitation at 488 nm. Scale bars: 1 mm. Immunostaining of explanted TA muscle 30 days after injection of **E**, 2D culture‐derived or **F**, ESM‐derived myogenic cells; eGFP: green epifluorescence, anti‐GFP: white, f‐actin: magenta, nuclei: blue; graft area marked by dotted line is denoted by eGFP^+^‐cells Scale bars: 100 µm. **G**, Quantification of eGFP^+^ donor myofibers per muscle section in injured area (n = 3 [2D], n = 5 [ESM] animals from two experimental series). *P < .05 by Mann‐Whitney test. **H**, Representative images of a eGFP^+^ Pax7^+^ cell in satellite cell position (Arrow) 30 days after ESM‐derived cell injection. eGFP: green epifluorescence, Pax7: white, f‐actin: magenta, nuclei: blue. Scale bar: 50 µm

To fully assess the autonomous regenerative potential of the myogenic cells in the ESM, we assessed whether functional satellite cells could be isolated from ESM using a protocol similar to that for the isolation of satellite cells from skeletal muscle.^42^ To unequivocally identify the progeny of ESM‐derived satellite cells in vitro and in vivo we constructed ESM from eGFP‐transgenic rats.^28^ To isolate the satellite cells, we dispersed the ESMs enzymatically and separated small, round cells from remaining myofibers by differential straining (Figure 7A). The isolated mononucleated cells contained 17 ± 5% (n = 4) Pax7^+^ cells without evidence of any remaining myofibers (Figure 7A). Interestingly, ESM‐isolated cells could be utilized to construct “2nd generation ESM” with similar functional performance as the parental “1st generation ESM” (Figure 7B) indicating high myogenic potential of the isolates. The observation of Pax7^+^ adjacent to the newly formed skeletal muscle provided evidence for the reconstruction of satellite cell niches also in the “2nd generation ESM” with similar numbers of Pax7^+^ cells (1st generation: 0.18 ± 0.03 Pax7^+^ nuclei per myonucleus, 2nd generation: 0.22 ± 0.02 Pax7^+^ nuclei per myonucleus, n = 3; Figure 7C).

To investigate the potential of ESM‐derived satellite cells in vivo we established a CTX injury model in immunocompromised RNU (nude) rats. In this model we first blocked endogenous regeneration by irradiation. As anticipated, muscle regeneration after CTX‐injury was greatly reduced in irradiated muscle (Figure S3). Three days after injury eGFP^+^ ESM‐derived cells were injected and could be traced by their epifluorescence even macroscopically (Figure 7D). In parallel we injected cells from age‐matched 2D cultures (14 days; 2D and ESM were from the same muscle cell isolations). Despite the presence of eGFP^+^‐cells derived from 2D culture in the muscle after 30 days, we did not observe muscle regeneration (Figure 7E). Conversely, we observed substantial remuscularization ‐ on average 17 ± 2 eGFP^+^ donor muscle fibers ‐ in rats (n = 5) injected with ESM‐derived cells (Figure 7F, G). Staining for Pax7 identified eGFP^+^ donor cells in a satellite cell position after implantation (Figure 7H).

## 4 DISCUSSION

Satellite cells are the regenerative components in skeletal muscle and typically located in morphologically defined satellite cell niches.^4^, ^5^ Here, we describe a tissue engineering modality capable of retaining satellite cell “anatomy” and functionality, both could not be readily anticipated given the commonly observed rapid loss of muscle stem cells properties in standard 2D cultures.^10^, ^11^, ^43^ In addition, we demonstrate that ESMs exhibit many properties of native skeletal muscle, including (i) morphological features of a syncytium composed of fused myocytes, (ii) basal contractile performance reminiscent of skeletal muscle (positive force‐frequency‐ and length‐force‐relationships, tetanic contractions), and (iii) responsiveness to pharmacological agents targeting the nicotinergic‐acetylcholine receptors.

The observation that satellite cells with true muscle stem cell properties 1, 11, 44, 45, 46, 47 can be preserved in ESM appears particularly intriguing and supports earlier studies on the regeneration potential of tissue engineered muscle.^25^ These properties include expression of characteristic proteins (Pax7, caveolin‐1, m‐cadherin), localization in a typical anatomical niche underneath the basal lamina and adjacent to well differentiated myotubes in a non‐proliferating state with the propensity for cell cycle activation, myogenic differentiation and functional regeneration upon injury. We have confirmed the regenerative capacity of the engineered satellite cell niche in the following in vitro and in vivo models: (1) severe muscle damage by cardiotoxin (CTX) administration, (2) mild mechanical (“crush”) muscle damage, (3) re‐isolation of functional satellite cells from “1st generation” ESMs for the generation of “2nd generation” ESMs, and (4) regeneration of CTX‐induced skeletal muscle damage in nude rats with impaired endogenous regeneration capacity.

Intramuscular CTX‐injection is an established method for the induction of skeletal muscle damage in vivo.^48^, ^49^ We adapted this model to induce ESM damage and found that CTX at 25 µg/ml for 24 hours induced marked myotube damage resulting in full contractile failure of ESM. An interesting observation was that this protocol did not destroy the reengineered satellite cell niche in ESM as evidenced by largely unaltered Pax7^+^ transcript abundance and a reduced, but not eliminated Pax7^+^ cell count. This data is on the one hand in line with the reported high susceptibility of differentiated muscle cells to CTX 6 and suggests on the other hand that satellite cells entail an intrinsic resistance to CTX. Based on these observations, we hypothesized that the unaffected Pax7‐positive satellite cells are activated upon injury to regenerate ESM. We tested this hypothesis by making use of an irradiation protocol typically applied in vivo to deplete/inactivate the satellite stem cell pool 35, 36 and could indeed observe that the endogenous regenerative capacity of ESM in response to CTX‐injury was abolished by irradiation, which advances earlier studies by demonstrating that muscle regeneration in vitro is indeed driven by satellite cells reentering the cell cycle.^25^ In line with a recent report 38 and findings in other organ models,^50^ macrophages may also play a crucial role in skeletal muscle regeneration. Given the muscle origin of the cells used for ESM generation and the presence of macrophages in most, if not all primary cell isolates (refer for example to primary cells isolated from the heart and their use in tissue engineering 30), we anticipated and finally confirmed the presence of macrophages in ESM. Future studies will have to address the heterologous cell content dynamics of ESM under homeostatic and injury conditions in more detail to better understand the contribution of specific cell types. The observation that the regenerative response in ESM can be enhanced or decreased by pharmacological modulation is in our view exciting as it provides early proof‐of‐concept for a utilization in phenotypic drug screens.

We substantiated the role of Pax7^+^ cells in muscle repair by making use of a novel in vitro model of spatially defined “crush injury”. In this series of experiments locally confined activation of Pax7^+^ muscle progenitors/satellite cells provided evidence for the specificity of the proposed muscle repair mechanism. Collectively, our findings are in agreement with the concept that ESMs resemble *bona fide* skeletal muscle surrogates, which encompasses the capacity for autonomous structural and functional regeneration via satellite cell activation.

Finally, we were able to re‐isolate functional satellite cells from ESM in a similar way as demonstrated for satellite cell isolation from native muscle.^42^ These data suggest that ESMs may be further exploited as a in vitro satellite cell expansion/preservation tool for potential therapeutic applications. This assumption is supported by implantation experiments whereby the injection of ESM‐derived isolates into injured and by irradiation regeneration‐incompetent muscle resulted in considerable remuscularization. As the ESM‐derived isolates also contained non‐muscle cells we cannot exclude that those supported engraftment of muscle stem cells. The observation that injections of 2D cultured muscle cells, similarly isolated as the cells for the ESM cultures, did not result in any palpable regeneration corroborates that the ESM culture format provided a unique environment for the maintenance of a functional satellite cell niche in vitro.

Notably, the majority of satellite cells were quiescent in ESM under basal conditions. This is remarkable because during isolation of primary satellite cells they are typically activated to proliferate and progress along the default myogenic differentiation pathway.^51^ The observation of predominantly quiescent Pax7^+^ cells in ESM may be explained by the presence of a population of Pax7^+^/MyoD^+^ “reserve cells” 52, 53, 54, 55, 56, 57 in the original ESM reconstitution mixture. Alternatively, there may have been a selection bias for specific stem/progenitor subpopulations with the capacity for symmetric and asymmetric self‐renewal in the primary skeletal muscle cell isolate.^48^ Further studies utilizing transgenic mouse models (eg Myf5‐Cre/ROSA‐YFP, NES‐GFP/Myf5^nLacZ/+^ 48, 56, 57) may be instructive to address whether the engineered satellite cell niche in ESM is indeed populated by a distinct myogenic cell subpopulation.

In vivo, the satellite cell niche is influenced by a variety of factors including growth factors, extracellular matrix (ECM), cell‐cell interactions, and likely other as of yet unidentified biochemical and biophysical cues.^4^ We aimed to control for the influence of extracellular matrix and growth factors on satellite cell function by parallel culture of the primary skeletal muscle cell isolate under conventional 2D conditions using the same extracellular matrix (ie rat tail collagen type 1 and Matrigel^®^) as adhesive growth substrate and medium as in 3D culture (ESM). The observation that these matrix and medium factors alone did not suffice to preserve Pax7^+^ cells suggests that other factors may be more relevant. Tissue elasticity was discovered to control stem cell properties in satellite cells.^12^ In this study, growth of satellite cells on a substrate with tissue‐like elasticity (12 kPa) was sufficient to retain a high degree of the cells regenerative potential. Interestingly, the elastic modulus of a day 12 ESM is in a similar range (9.5 kPa) and thus it is tempting to speculate that the tissue‐like biomechanical properties of ESM contributed to preservation of satellite cell function in our model. However, further dissection of biochemical and mechanical cues as demonstrated by a recent elegant study from Quarta et al is required to fully define and control the satellite cell niche in ESM.^13^

We conclude that the ESM technology enables robust engineering of skeletal muscle with functional satellite cell niches in vitro. Importantly, regenerative potential could be documented morphologically, but also by very sensitive means of isometric force measurements. We feel that partial and full recovery of tissue functionality after injury, as observed in the CTX and the novel “crush” injury model, as well as complete loss of regeneration after irradiation provide strong evidence for the “physiological” functionality of the engineered satellite cell niche in ESM. Given these findings we propose that the ESM model may represent a versatile tool to (1) further dissect satellite cell biology, (2) establish skeletal muscle disease models in vitro for drug development, and (3) provide potential therapeutic satellite cells for cell‐based skeletal muscle repair. The availability of human pluripotent stem cells and methods for their directed myoblast differentiation 58, 59 will facilitate the translation of the reported rat to a human model. In fact, alternative models of engineered human skeletal muscle using the forced expression of Pax7 60 or directed multi‐lineage differentiation in induced pluripotent stem cells 61 have recently been reported. These advances will be important for applications in screens for regeneration inducing compounds and studies of cell based muscle repair. A challenge will be to establish the cellular complexity of *bona fide* skeletal muscle, which may be achieved by a further refinement of multi‐lineage stem cell differentiation or the optimization of primary cell isolation from human skeletal muscle.

## CONFLICT OF INTEREST

Authors declare no conflict of interest.

## AUTHOR CONTRIBUTIONS

M. Tiburcy designed research, performed research, analyzed data, and wrote the paper, A. Markov, L. K. Kraemer, P. Christalla performed research and analyzed data, M. Rave‐Fraenk, H. J. Fischer, and H. M. Reichardt contributed material and experimental tools, Wolfram‐Hubertus Zimmermann designed research and edited paper.

## References

[1] Collins CA , Olsen I , Zammit PS , et al. Stem cell function, self‐renewal, and behavioral heterogeneity of cells from the adult muscle satellite cell niche. Cell. 2005;122:289‐301. 16051152 10.1016/j.cell.2005.05.010

[2] Lepper C , Partridge TA , Fan CM . An absolute requirement for Pax7‐positive satellite cells in acute injury‐induced skeletal muscle regeneration. Development. 2011;138:3639‐3646. 21828092 10.1242/dev.067595 PMC3152922

[3] Sambasivan R , Yao R , Kissenpfennig A , et al. Pax7‐expressing satellite cells are indispensable for adult skeletal muscle regeneration. Development. 2011;138:3647‐3656. 21828093 10.1242/dev.067587

[4] Kuang S , Gillespie MA , Rudnicki MA . Niche regulation of muscle satellite cell self‐renewal and differentiation. Cell Stem Cell. 2008;2:22‐31. 18371418 10.1016/j.stem.2007.12.012

[5] Morgan JE , Partridge TA . Muscle satellite cells. Int J Biochem Cell Biol. 2003;35:1151‐1156. 12757751 10.1016/s1357-2725(03)00042-6

[6] Charge SB , Rudnicki MA . Cellular and molecular regulation of muscle regeneration. Physiol Rev. 2004;84:209‐238. 14715915 10.1152/physrev.00019.2003

[7] Tedesco FS , Dellavalle A , Diaz‐Manera J , Messina G , Cossu G . Repairing skeletal muscle: regenerative potential of skeletal muscle stem cells. J Clin Invest. 2010;120:11‐19. 20051632 10.1172/JCI40373 PMC2798695

[8] Beauchamp JR , Morgan JE , Pagel CN , Partridge TA . Dynamics of myoblast transplantation reveal a discrete minority of precursors with stem cell‐like properties as the myogenic source. J Cell Biol. 1999;144:1113‐1122. 10087257 10.1083/jcb.144.6.1113 PMC2150577

[9] Mendell JR , Kissel JT , Amato AA , et al. Myoblast transfer in the treatment of Duchenne's muscular dystrophy. N Engl J Med. 1995;333:832‐838. 7651473 10.1056/NEJM199509283331303

[10] Brack AS , Conboy IM , Conboy MJ , Shen J , Rando TA . A temporal switch from notch to Wnt signaling in muscle stem cells is necessary for normal adult myogenesis. Cell Stem Cell. 2008;2:50‐59. 18371421 10.1016/j.stem.2007.10.006

[11] Montarras D , Morgan J , Collins C , et al. Direct isolation of satellite cells for skeletal muscle regeneration. Science. 2005;309:2064‐2067. 16141372 10.1126/science.1114758

[12] Gilbert PM , Havenstrite KL , Magnusson KE , et al. Substrate elasticity regulates skeletal muscle stem cell self‐renewal in culture. Science. 2010;329:1078‐1081. 20647425 10.1126/science.1191035 PMC2929271

[13] Quarta M , Brett JO , DiMarco R , et al. An artificial niche preserves the quiescence of muscle stem cells and enhances their therapeutic efficacy. Nat Biotechnol. 2016;34:752‐759. 27240197 10.1038/nbt.3576 PMC4942359

[14] Pruller J , Mannhardt I , Eschenhagen T , Zammit PS , Figeac N . Satellite cells delivered in their niche efficiently generate functional myotubes in three‐dimensional cell culture. PLoS ONE. 2018;13:e0202574. 30222770 10.1371/journal.pone.0202574 PMC6141091

[15] Christalla P , Hudson JE , Zimmermann WH . The cardiogenic niche as a fundamental building block of engineered myocardium. Cells Tissues Organs. 2011. 10.1159/000331407 21996934

[16] Vunjak‐Novakovic G , Scadden DT . Biomimetic platforms for human stem cell research. Cell Stem Cell. 2011;8:252‐261. 21362565 10.1016/j.stem.2011.02.014 PMC3048803

[17] Lutolf MP , Gilbert PM , Blau HM . Designing materials to direct stem‐cell fate. Nature. 2009;462:433‐441. 19940913 10.1038/nature08602 PMC2908011

[18] Griffith LG , Swartz MA . Capturing complex 3D tissue physiology in vitro. Nat Rev Mol Cell Biol. 2006;7:211‐224. 16496023 10.1038/nrm1858

[19] Vandenburgh HH , Karlisch P , Farr L . Maintenance of highly contractile tissue‐cultured avian skeletal myotubes in collagen gel. Vitro Cell Dev Biol. 1988;24:166‐174. 10.1007/BF02623542 3350785

[20] Powell C , Shansky J , Del Tatto M , et al. Tissue‐engineered human bioartificial muscles expressing a foreign recombinant protein for gene therapy. Hum Gene Ther. 1999;10:565‐577. 10094200 10.1089/10430349950018643

[21] Vandenburgh H , Shansky J , Benesch‐Lee F , et al. Drug‐screening platform based on the contractility of tissue‐engineered muscle. Muscle Nerve. 2008;37:438‐447. 18236465 10.1002/mus.20931

[22] Vandenburgh H , Shansky J , Benesch‐Lee F , et al. Automated drug screening with contractile muscle tissue engineered from dystrophic myoblasts. Faseb J. 2009;23:3325‐3334. 19487307 10.1096/fj.09-134411 PMC3236595

[23] Koffler J , Kaufman‐Francis K , Shandalov Y , et al. Improved vascular organization enhances functional integration of engineered skeletal muscle grafts. Proc Natl Acad Sci U S A. 2011;108:14789‐14794. 21878567 10.1073/pnas.1017825108 PMC3169163

[24] Madden L , Juhas M , Kraus WE , Truskey GA , Bursac N . Bioengineered human myobundles mimic clinical responses of skeletal muscle to drugs. eLife. 2015;4:e04885. 25575180 10.7554/eLife.04885 PMC4337710

[25] Juhas M , Engelmayr GC Jr , Fontanella AN , Palmer GM , Bursac N . Biomimetic engineered muscle with capacity for vascular integration and functional maturation in vivo. Proc Natl Acad Sci U S A. 2014;111:5508‐5513. 24706792 10.1073/pnas.1402723111 PMC3992675

[26] Juhas M , Bursac N . Roles of adherent myogenic cells and dynamic culture in engineered muscle function and maintenance of satellite cells. Biomaterials. 2014;35:9438‐9446. 25154662 10.1016/j.biomaterials.2014.07.035 PMC4157105

[27] Bursac N , Juhas M , Rando TA . Synergizing Engineering and Biology to Treat and Model Skeletal Muscle Injury and Disease. Annu Rev Biomed Eng. 2015;17:217‐242. 26643021 10.1146/annurev-bioeng-071114-040640 PMC4858326

[28] van den Brandt J , Wang D , Kwon SH , Heinkelein M , Reichardt HM . Lentivirally generated eGFP‐transgenic rats allow efficient cell tracking in vivo. Genesis. 2004;39:94‐99. 15170694 10.1002/gene.20037

[29] Naito H , Melnychenko I , Didie M , et al. Optimizing engineered heart tissue for therapeutic applications as surrogate heart muscle. Circulation. 2006;114:I72‐78. 16820649 10.1161/CIRCULATIONAHA.105.001560

[30] Zimmermann WH , Schneiderbanger K , Schubert P , et al. Tissue engineering of a differentiated cardiac muscle construct. Circ Res. 2002;90:223‐230. 11834716 10.1161/hh0202.103644

[31] Tiburcy M , Didie M , Boy O , et al. Terminal Differentiation, Advanced Organotypic Maturation, and Modeling of Hypertrophic Growth in Engineered Heart Tissue. Circ Res. 2011. 10.1161/CIRCRESAHA.111.251843 21921264

[32] Zimmermann WH , Fink C , Kralisch D , Remmers U , Weil J , Eschenhagen T . Three‐dimensional engineered heart tissue from neonatal rat cardiac myocytes. Biotechnol Bioeng. 2000;68:106‐114. 10699878

[33] de Haan A , de Ruiter CJ , Lind A , Sargeant AJ . Growth‐related change in specific force but not in specific power of rat fast skeletal muscle. Exp Physiol. 1992;77:505‐508. 1632958 10.1113/expphysiol.1992.sp003611

[34] Drachman DB , Johnston DM . Development of a mammalian fast muscle: dynamic and biochemical properties correlated. J Physiol. 1973;234:29‐42. 4271901 10.1113/jphysiol.1973.sp010332 PMC1350649

[35] Heslop L , Morgan JE , Partridge TA . Evidence for a myogenic stem cell that is exhausted in dystrophic muscle. J Cell Sci. 2000;113(Pt 12):2299‐2308. 10825301 10.1242/jcs.113.12.2299

[36] Rosenblatt JD , Parry DJ . Adaptation of rat extensor digitorum longus muscle to gamma irradiation and overload. Pflugers Arch. 1993;423:255‐264. 8321629 10.1007/BF00374404

[37] Arnold L , Henry A , Poron F , et al. Inflammatory monocytes recruited after skeletal muscle injury switch into antiinflammatory macrophages to support myogenesis. J Exp Med. 2007;204:1057‐1069. 17485518 10.1084/jem.20070075 PMC2118577

[38] Juhas M , Abutaleb N , Wang JT , et al. Incorporation of macrophages into engineered skeletal muscle enables enhanced muscle regeneration. Nat Biomed Eng. 2018;2:942‐954. 30581652 10.1038/s41551-018-0290-2 PMC6296488

[39] Conboy IM , Rando TA . The regulation of Notch signaling controls satellite cell activation and cell fate determination in postnatal myogenesis. Dev Cell. 2002;3:397‐409. 12361602 10.1016/s1534-5807(02)00254-x

[40] Jones AE , Price FD , Le Grand F , et al. Wnt/beta‐catenin controls follistatin signalling to regulate satellite cell myogenic potential. Skelet Muscle. 2015;5:14. 25949788 10.1186/s13395-015-0038-6 PMC4421991

[41] Otto A , Schmidt C , Luke G , et al. Canonical Wnt signalling induces satellite‐cell proliferation during adult skeletal muscle regeneration. J Cell Sci. 2008;121:2939‐2950. 18697834 10.1242/jcs.026534

[42] Conboy MJ , Conboy IM . Preparation of adult muscle fiber‐associated stem/precursor cells. Methods Mol Biol. 2010;621:149‐163. 20405365 10.1007/978-1-60761-063-2_10

[43] Ito N , Shimizu N , Tanaka H , Takeda S . Enhancement of Satellite Cell Transplantation Efficiency by Leukemia Inhibitory Factor. J Neuromuscul Dis. 2016;3:201‐207. 27854222 10.3233/JND-160156 PMC5271580

[44] Relaix F , Montarras D , Zaffran S , et al. Pax3 and Pax7 have distinct and overlapping functions in adult muscle progenitor cells. J Cell Biol. 2006;172:91‐102. 16380438 10.1083/jcb.200508044 PMC2063537

[45] Snow MH . An autoradiographic study of satellite cell differentiation into regenerating myotubes following transplantation of muscles in young rats. Cell Tissue Res. 1978;186:535‐540. 627031 10.1007/BF00224941

[46] Sacco A , Doyonnas R , Kraft P , Vitorovic S , Blau HM . Self‐renewal and expansion of single transplanted muscle stem cells. Nature. 2008;456:502‐506. 18806774 10.1038/nature07384 PMC2919355

[47] Gnocchi VF , White RB , Ono Y , Ellis JA , Zammit PS . Further characterisation of the molecular signature of quiescent and activated mouse muscle satellite cells. PLoS ONE. 2009;4:e5205. 19370151 10.1371/journal.pone.0005205 PMC2666265

[48] Kuang S , Kuroda K , Le Grand F , Rudnicki MA . Asymmetric self‐renewal and commitment of satellite stem cells in muscle. Cell. 2007;129:999‐1010. 17540178 10.1016/j.cell.2007.03.044 PMC2718740

[49] Carlson ME , Hsu M , Conboy IM . Imbalance between pSmad3 and Notch induces CDK inhibitors in old muscle stem cells. Nature. 2008;454:528‐532. 18552838 10.1038/nature07034 PMC7761661

[50] Aurora AB , Porrello ER , Tan W , et al. Macrophages are required for neonatal heart regeneration. J Clin Invest. 2014;124:1382‐1392. 24569380 10.1172/JCI72181 PMC3938260

[51] Mourikis P , Sambasivan R , Castel D , Rocheteau P , Bizzarro V , Tajbakhsh S . A Critical Requirement for Notch Signaling in Maintenance of the Quiescent Skeletal Muscle Stem Cell State. Stem Cells. 2011. 10.1002/stem.775 22069237

[52] Zammit PS , Relaix F , Nagata Y , et al. Pax7 and myogenic progression in skeletal muscle satellite cells. J Cell Sci. 2006;119:1824‐1832. 16608873 10.1242/jcs.02908

[53] Shea KL , Xiang W , LaPorta VS , et al. Sprouty1 regulates reversible quiescence of a self‐renewing adult muscle stem cell pool during regeneration. Cell Stem Cell. 2010;6:117‐129. 20144785 10.1016/j.stem.2009.12.015 PMC2846417

[54] Horsley V , Friday BB , Matteson S , Kegley KM , Gephart J , Pavlath GK . Regulation of the growth of multinucleated muscle cells by an NFATC2‐dependent pathway. J Cell Biol. 2001;153:329‐338. 11309414 10.1083/jcb.153.2.329 PMC2169453

[55] Friday BB , Pavlath GK . A calcineurin‐ and NFAT‐dependent pathway regulates Myf5 gene expression in skeletal muscle reserve cells. J Cell Sci. 2001;114:303‐310. 11148132 10.1242/jcs.114.2.303

[56] Day K , Shefer G , Shearer A , Yablonka‐Reuveni Z . The depletion of skeletal muscle satellite cells with age is concomitant with reduced capacity of single progenitors to produce reserve progeny. Dev Biol. 2010;340:330‐343. 20079729 10.1016/j.ydbio.2010.01.006 PMC2854302

[57] Day K , Shefer G , Richardson JB , Enikolopov G , Yablonka‐Reuveni Z . Nestin‐GFP reporter expression defines the quiescent state of skeletal muscle satellite cells. Dev Biol. 2007;304:246‐259. 17239845 10.1016/j.ydbio.2006.12.026 PMC1888564

[58] Chal J , Al Tanoury Z , Hestin M , et al. Generation of human muscle fibers and satellite‐like cells from human pluripotent stem cells in vitro. Nat Protoc. 2016;11:1833‐1850. 27583644 10.1038/nprot.2016.110

[59] van der Wal E , Herrero‐Hernandez P , Wan R ,, et al. Large‐Scale Expansion of Human iPSC‐Derived Skeletal Muscle Cells for Disease Modeling and Cell‐Based Therapeutic Strategies. Stem Cell Reports. 2018;10:1975‐1990. 29731431 10.1016/j.stemcr.2018.04.002 PMC5993675

[60] Rao L , Qian Y , Khodabukus A , Ribar T , Bursac N . Engineering human pluripotent stem cells into a functional skeletal muscle tissue. Nat Commun. 2018;9:126. 29317646 10.1038/s41467-017-02636-4 PMC5760720

[61] Maffioletti SM , Sarcar S , Henderson ABH , et al. Three‐dimensional human iPSC‐derived artificial skeletal muscles model muscular dystrophies and enable multilineage tissue engineering. Cell Rep. 2018;23:899‐908. 29669293 10.1016/j.celrep.2018.03.091 PMC5917451

