# Supplementary material for: Regeneration competent satellite cell niches in rat engineered skeletal muscle
Source: FASEB Bioadv. 2019 Nov 13;1(12):731–46. doi: 10.1096/fba.2019-00013 (PMC6996344; doi:10.1096/fba.2019-00013)

SUPPLEMENTAL DATA

Regeneration competent satellite cell niches in rat Engineered Skeletal Muscle

Malte Tiburcy[1,2], Alex Markov[1,2], Lena K. Kraemer[1,2], Peter Christalla[1], Margret Rave-Fraenk[3], Henrike J. Fischer[4], Holger M. Reichardt[4], Wolfram-Hubertus Zimmermann[1,2]

[1]Institute of Pharmacology and Toxicology, Heart Research Center Göttingen, Georg-August University Göttingen, Robert-Koch-Str. 40, 37075 Göttingen, Germany; [2]DZHK (German Center for Cardiovascular Research), partner site Göttingen; [3]Department of Radiation Therapy and Radiooncology, Georg-August University Göttingen, Robert-Koch-Str. 40, 37075 Göttingen, Germany; [4]Institute for Cellular and Molecular Immunology, Georg-August University Göttingen, Humboldtallee 34, 37073 Göttingen, Germany

Supplemental legends

Supplemental Figure 1. Characterization of primary muscle isolates. Flow cytometry of primary skeletal muscle cell isolates after 5 day expansion *in vitro*. (**A**) Representative plot for myogenic markers Pax7 (left panel) and desmin (right panel). (**B**) Representative plot for macrophage markers ED1 (CD68, left panel) and ED2 (CD163, right panel).

Supplemental Figure 2. ESM regeneration is abolished after irradiation and supported by macrophages. (**A**) Immunostaining of day 14 control ESM and ESM after cardiotoxin (CTX) treatment at day 14 and day 21 with (top panel) and without irradiation (30 Gy, lower panel); Pax7: white, f-actin: green, Ki67: magenta, nuclei: blue; Scale bars: 50 µm. (**B**) Immunostaining for ED1 (CD68, top panel) and ED2 (CD163, lower panel) macrophages (highlighted by arrows) in day 14 control ESM and ESM after cardiotoxin (CTX) treatment at day 14 and day 21; ED1 or ED2: magenta, f-actin: green, nuclei: blue; Scale bars: 50 µm.

Supplemental Figure 3. ESM regeneration is reduced by inhibiting Notch signaling. (**A**) Immunostaining of day 14 control ESM and ESM after cardiotoxin (CTX) treatment at day 14 and day 21 in presence of 0.1% DMSO (top panel) or 0.5 µM Gamma-secretase inhibitor (GSI, lower panel); Pax7: white, f-actin: green, , nuclei: blue. Scale bars: 50 µm.

Supplemental Figure 4. Impaired regeneration in irradiated skeletal muscle. Skeletal muscle damage was induced by cardiotoxin injection (100 µl of 20 µmol/L solution) in

irradiated (40 Gy) and non-irradiated tibialis muscle (TA) of nude rats. HE staining was performed 3 or 33 days after cardiotoxin injury as indicated. Scale bars: 100 µm

Movie S1: Fibrillation and spontaneous synchronized contractions in a day 12 ESM free-floating in Tyrode's solution.

Movie S2: Projection of ESM z-axis sections (culture day 12). Staining for actin (green) and nuclei (blue). Z-stack images were acquired with a Leica TCS LSI macro confocal microscope.

Supplemental Figure 1

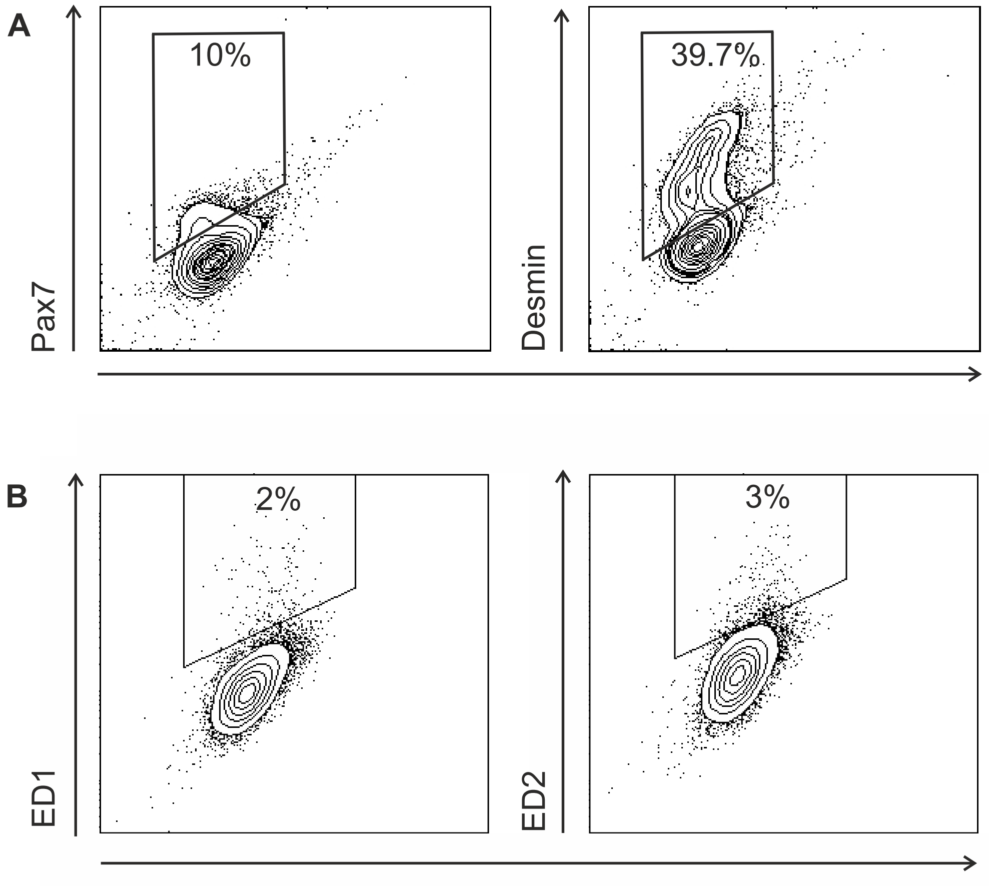

Supplemental Figure 2

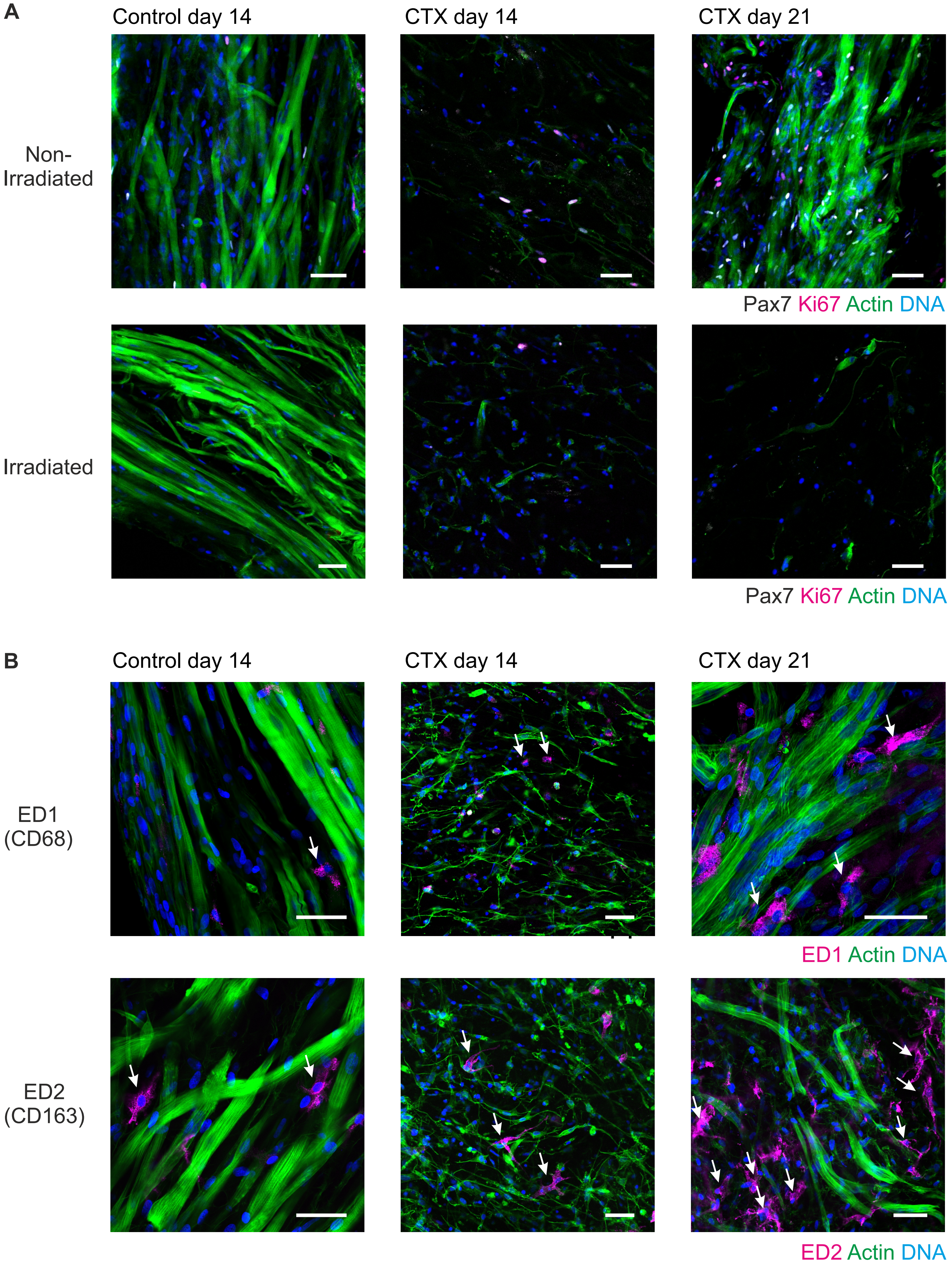

Supplemental Figure 3

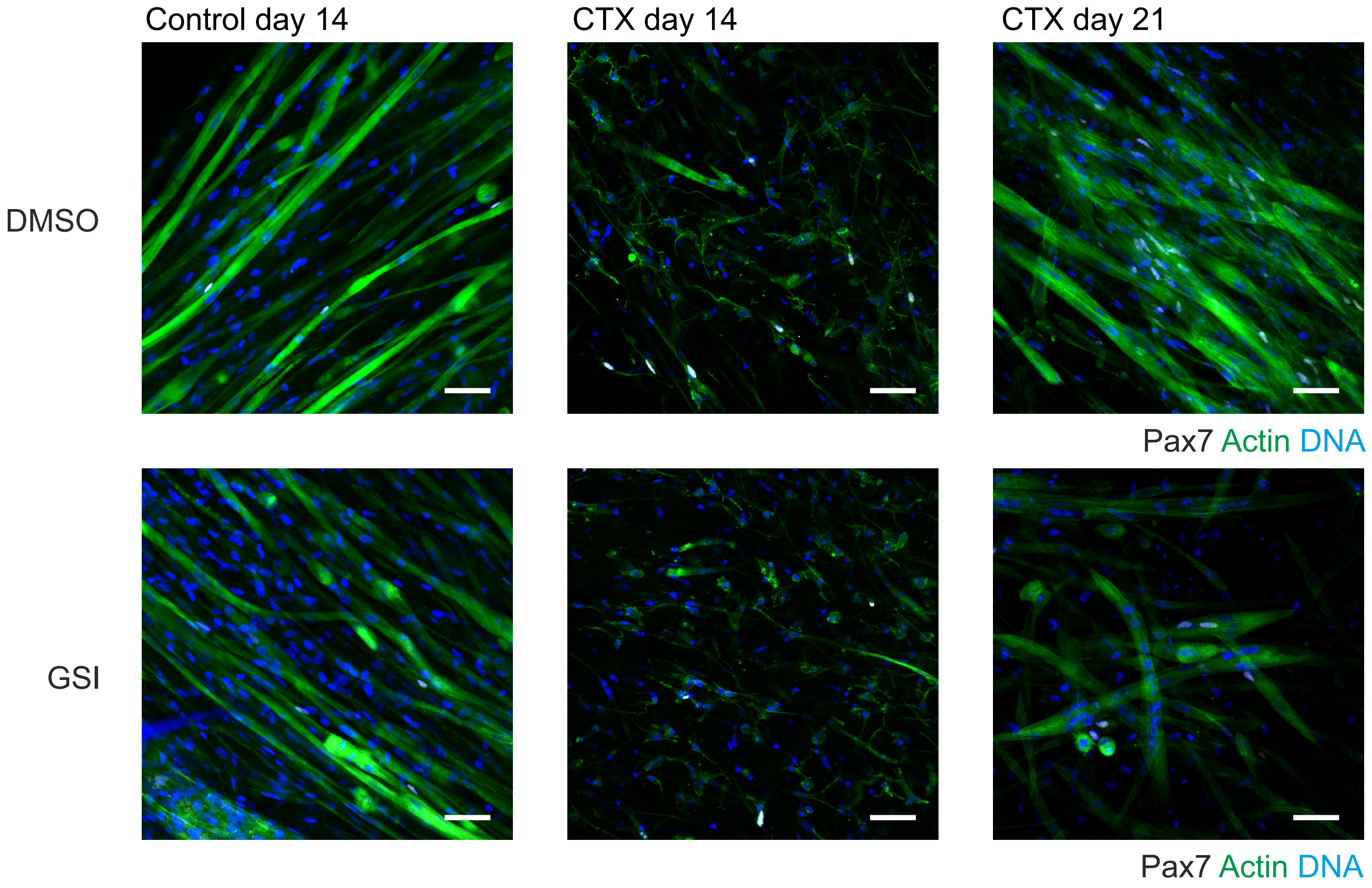

Supplemental Figure 4

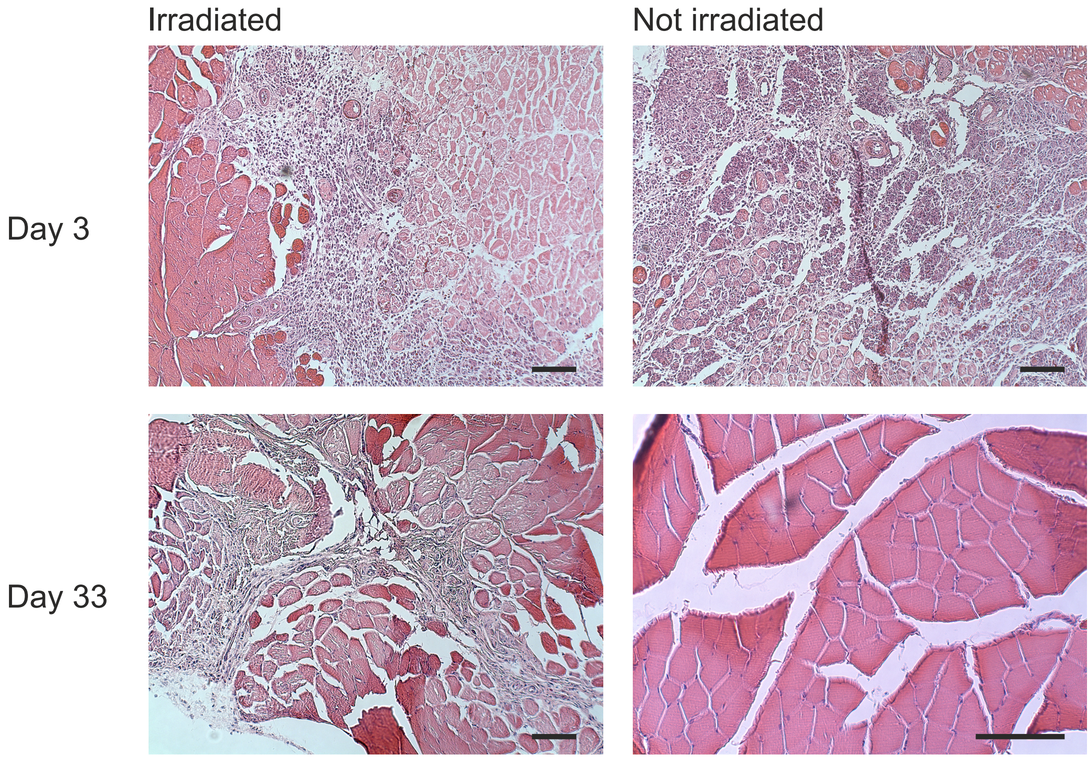

Supplement: Supplementary file 1 [file FBA2-1-731-s001.pdf]
